# Is deployment of trained nurses to rural villages a remedy for the low skilled birth attendance in Ethiopia? *A cluster randomized-controlled community trial*

**DOI:** 10.1371/journal.pone.0204986

**Published:** 2018-10-12

**Authors:** Taddese Alemu Zerfu, Henok Taddese, Tariku Nigatu, Girma Tenkolu, Dina Neelofur Khan, Sibhatu Biadgilign, Amare Deribew

**Affiliations:** 1 Maternal and Child Well Being Unit, African Population and Health Research Center, Nairobi, Kenya; 2 College of Health and Medical Sciences, Dilla University, Dilla, Ethiopia; 3 Institute of Public Heath, University of Gondar, Gondar, Ethiopia; 4 UNDP/UNFPA/UNICEF/WHO/World Bank Special Programme of Research, Development and Research Training in Human Reproduction (HRP), Department of Reproductive Health and Research, WHO, Geneva, Switzerland; 5 Public Health Research Consultant, Addis Ababa, Ethiopia; 6 St. Paul Millennium Medical College, Addis Ababa, Ethiopia; Management Sciences for Health, ETHIOPIA

## Abstract

**Background:**

Low coverage of Skilled Birth Attendance (SBA) is one of the major drivers of maternal mortality in many low- and middle-income countries (LMICs) including Ethiopia. We conducted a cluster-randomized controlled community trial to assess the effect of deploying trained community based nurses to rural communities on the uptake levels of SBA in Ethiopia.

**Methods:**

A three-arm, parallel groups, cluster-randomized community trial was conducted to assess the effect of deploying trained community based reproductive health nurses (CORN) on the uptake of SBA services. A total of 282 villages were randomly selected and assigned to a control arm (n = 94) or 1 of 2 treatment arms (n = 94 each). The treatment groups differed by where these new service providers were deployed, a health post (HP) or health center (HC). Baseline and end line surveys were conducted to document and measure the effects of the intervention. Program impacts on SBA coverage were calculated using difference‐in‐difference (DID) analysis.

**Results:**

After nine months of intervention, the coverage of SBA services increased significantly by 81.1% (from 24.61 to 44.59) in the HP based intervention arm, and by 122.9% (from 16.41 to 36.59) in the HC arm, respectively (p <0.01). Conversely, a small and non-significant (2%) decline in SBA coverage were observed in the control arm (P >0.05). The DID estimate indicated a net increase in SBA coverage of 21.32 and 20.52 percentage points (PP) across the HP and HC based intervention arms, respectively (p < 0.001).

**Conclusions:**

Deployment of trained reproductive health nurses to rural communities in Ethiopia significantly improved utilization of SBA services. Therefore; in similar low income settings where coverage of SBA services is very low, deployment of trained community based nurses to grassroots level could potentiate rapid service uptake. Additional cost-effectiveness and validation studies at various setups are required, before scale-up of the innovation, however.

**Trial registration:**

clinicaltrails.gov NCT02501252.

## Introduction

Even if the global progress has been suboptimal, maternal and child mortality rates have showed a steady declined over the last four decades [1– 4]. Today, all over the world, particularly in poor income settings, millions of women and newborns, continues to die from complications related to pregnancy or poor obstetric care at birth [5]. For every woman who dies, 30 to 50 women will further suffer injury, infection, or disease and obstetric complications, many of which are leading causes of death and disability amongst reproductive age women in low and middle income countries(LMICs) [5–7].

Improving the quality of obstetric intra-partum care could save an estimated 113, 000 maternal deaths, 531 000 stillbirths, and 325 million neonatal deaths annually by 2020 [8]. Similarly, increasing the coverage and quality of preconception, antenatal, intra-partum, and postnatal interventions by 2025 could avert an estimated 71% of neonatal deaths, 33% of stillbirths, and 54% of maternal deaths per year [8,9]. The most dramatic improvements to averting neonatal deaths can be made through interventions delivered during labor, birth and the immediate postnatal period [8].

Ethiopia has shown remarkable progress in the reduction of maternal and child mortality rates during the past 25 years [10–12]. The national mortality ratio declined by 67% (from 1250 deaths per 100,000 live births in 1990 to 420 in 2015), which is slightly below the MDG target [13–17]. Nevertheless, the total number of maternal and neonatal deaths and mortality rates remain high with complex and interwoven risk factors [18–21]. Low coverage of facility-based deliveries, poor competence of providers, and poor quality of care and referral system are key contributing factors for the unacceptably high mortality rates [17–24]. Recent estimates shows that in rural parts of Ethiopia, which account 85% of the national population, only 16% childbirths are attended by skilled providers [25, 26].

To improve access to basic health care services and reduce the unacceptably high level of maternal and child mortality, in 2003, the government of Ethiopia launched an innovative community-based health service delivery strategy, the Health Extension Program (HEP) [27, 28]. The HEP involves deployment of trained and salaried female frontline health workers who can provide basic primary health care services, but not skilled care services including delivery, at community level. Since the program inception, more than 35,000 Health Extension Workers (HEW) have been deployed to all kebele (lowest administrative unit having a total population of 5000). The program includes 17 essential health service packages under four major program areas: hygiene and environmental sanitation, disease prevention and control, and family health services and health promotion and communication [27,28].

Though the HEP has contributed to the improvement of women’s utilization of family planning, antenatal care (ANC) and HIV testing, evidence is accumulating on the limitations of the program in addressing utilization of skilled maternity services. So far, the HEP did not contribute significantly for change in the reduction of maternal and neonatal deaths [29 –31].

According to studies related to HEP, leading barriers to effective implementation of the program were geared towards the HEW: high turnover [32,33] and frequent absence from work [32,34], limited technical skill, poor interaction with the community [30,31], and lack of social mobilization skills [35–37]. The objective of this study was to assess the effect of deploying trained and CORNs on the coverage of SBA in rural Ethiopia.

## Methods

### Study settings

A brief description of the same is given here as follows; the study was conducted in three rural districts of Gedeo Zone in Southern, Nations, Nationalities and Peoples (SNNP) region of Ethiopia from October 2014 to November 2016. Six *Kebeles* (smallest administrative units) in each district were randomly assigned to one of the three arms of the study. Dilla University Hospital is a referral centre for the catchment population surrounding the health facilities. Details on the methods of the present study is given elsewhere in a published methodology [38].

### Study design

The study employed a three arm parallel-groups, cluster-randomized, controlled trial with balanced randomization (1:1). A total of 18 kebeles having 282 villages were selected and randomly assigned to the three arms. Each village has an estimated 350–400 total population; of whom 10–12 are pregnant women. Accordingly, trained nurses (CORN) were deployed to provide similar services, while positioned either at HP or HC. In Arm 1, CORNs were deployed to health posts to provide skilled maternal and newborn care services to villages (health post-based intervention); whereas in Arm 2, they were to providing similar services positioned at health centers (health center-based intervention). In the control arm (Arm 3), no change to current maternal and newborn health service provision was made.

In the Ethiopian context, Primary Health Care Unit (PHCU) forms the basic unit of the national health care system and comprises a health center and five satellite health posts that are expected to serves up to 25,000 and 5,000 catchment population, respectively. A health post is staffed by two HEWs that provide preventive and promotive health care, including healthy living and environmental sanitation, prevention of major public health diseases and epidemics, and mobilizing communities for health actions. Similarly, a health center serves as a first-level referral for health posts and provide curative care for a variety of common diseases. They also provide emergency surgery in selected areas [39].

CORNs are graduate nurses from an accredited higher learning institutions in Ethiopia. After recruitment for this study, CORNS received an additional four months intensive skill oriented training at Dilla University Hospital based on competency model. The competency based training of CORNs focused on basic and emergency obstetric care such as practical skills, problem solving, critical thinking and decision-making skills. On the other hand, health extension workers are female community health workers with one year of training focusing on 16 promotive and preventive health care packages [3].

### Participants

Eligible participants were all women who become pregnant (amenorrhic at least for three menstrual cycles and reported positive for human chorionic gonadotropin (hCG) urine test at the time of baseline data collection), those who lived in the study area for at least for six months and those who were willing to participate in the study. Exclusion criteria were those who were non-pregnant during or before the study period.

### Interventions

The interventions consisted of provision of maternal, neonatal and child health care services by CORN based at either health centers or health posts. The interventions were conducted in four phases. After sensitizing relevant stakeholders and recruiting 16 nurses for intervention, a structured and target oriented skill based training was given for four months on SBA and other reproductive health (RH) care services. This was followed by a nine months intervention deploying the trained CORN in their respective arms based on the decision algorithm (published protocol). A total of twelve CORNs (six per each of the intervention arm) were deployed to provide skilled SBA services at grass root level.

According to the CORN decision algorithm, the first step was to educate and counsel mothers on the need for institutional delivery during home visit (Plan A). This is to encourage and clear misconceptions/information gaps/ so that mothers would use skilled institutional health services including: ANC, SBA, PNC and FP services. CORN also made a fortnightly home visits to ensure compliance of recommend and promised services. As such, if a women was found to be non-compliant, CORN provided SBA and other skilled MCH services at the nearby HP (Plan B). As a last option, if a mother is still non-compliant for either of plans A or B, they directly provided the service at home (Plan C). On the other hand, HEW in the control (non-intervention) kebeles used routine care models, in accordance with standard HEP practices.

### Data collection and measurement of the outcome

Baseline and endline data were collected by trained nurses and high school graduates during November to December 2014 and between February-March 2016, respectively. Socio-demographic variables, reproductive health characteristics, maternal health service utilization indicators (such as SBA, FP, FANC, PNC and PMTCT) were key variables traced in the surveys. Clinical assessments were conducted monthly during the follow-up period. To assess pregnancy and gestational weeks of pregnancy, obstetric history (the period of the Last Menstrual Period (LMP), a human chorionic gonadotropin (hCG) urine test [40,41] and Leopold’s Maneuvers of fundal palpation were used.

The primary outcome was the proportion of pregnant women who gave birth at health facility through the assistance of skilled care provider such as midwives, doctors or other trained professionals. Secondary outcomes included proportion of mothers who obtained skilled care from CORN at home, health post or health center.

### Data management and statistical analysis

Data were entered into Epi-Data software, cleaned, and analyzed using Statistical Package for Social Sciences (SPSS-20) and STATA 13.0 (Stata Corporation, College Station, TX). An intention-to-treat analysis was completed. To assess impacts of the intervention on SBA utilization, we used difference-in-difference (DID) estimates derived from simple differences of endline and baseline values and further difference from control differences. The DID is based on the differential effect of an intervention (treatment) on a ‘treatment arm’ versus a ‘control arm’. Hence, in our present analysis, we computed the effect of the intervention on SBA (outcome) by comparing the average change in mean proportion over time in the outcome variable for the intervention group, compared to the average change in mean proportion over nine months’ time for the control arm. These estimates examined the change in the program indicator variables between the baseline and endline surveys; differences in proportions for the HP and HC villages were estimated as the difference in the proportional change between the HP relative to the control group. A p-value of less than 0.05 was considered statistically significant. Reporting followed the CONSORT statement for reporting of cluster randomized controlled trials [42].

#### Ethics approval and consent to participate

The study received ethical approval from Dilla University Institutional Research Ethics Committee as well as the World Health Organization (WHO) Ethics committee. The study followed the CONSORT guideline. Written consent was obtained from the study participants.

## Results

### Baseline characteristics and attrition rate

A total of 2,147 and 2,142 women participated in the baseline and endline surveys respectively. The proportion of mothers with formal education (primary and above) was higher in the health center-based intervention villages (35.5%) compared to that of the health post-based intervention (23.9%) and the control villages (25.9%). Mean age of mothers at pregnancy was lower in the health post-based intervention villages (17.4 ± 2.2) than the mean age of pregnancy in the health center-based intervention (18.04± 2.2) and the control villages (18.1 ± 2.4) (Table 1).

**Table 1:** Mean baseline socio-demographic and reproductive characteristics of mothers in control and intervention villages in rural Gedeo, Southern Ethiopia^1^.

Variable	All	Control	CORN HP	CORN HC
Mothers, n	2147	717	711	719
HH size (n ± SD)	5**∙**78 ± 2**∙**14	5**∙**62 ± 1.99	5.96 ± 2.32	5.77 ± 2.06
HH income, ETB per month^2^ (n ± SD)	526.5 ± 13	731.2 ± 25	454.46 ± 20	366.7 ± 17
Parity (Total Number of deliveries)	4.16 ± 2.4	4.1 ± 2.2	4.18 ± 2.3	4.36 ± 2.6
Gravidity (Number of pregnancies)	4.33 ± 2.5	4.22 ± 2.4	4.31 ± 2.4	4.45 ± 2.6
Current age of the mother	28.3 ± 6.2	27.6 ± 5.7	27.9 ± 5.8	29.3± 6.8
Age of mother at first pregnancy	18.1 ± 2.4	18.9 ± 2.2	17.4 ± 2.2	18.04± 2.2
Ethnic group, *n (%)*				
Gedeo	2031 (94.6)	690 (96)	673 (94.7)	690 (95.9)
Non-Gedeo	116 (5.4)	29 (4.0)	38 (5.3)	29 (4.1)
Educational status, *n (%)*				
Unable to read and write	1453 (67.7)	499 (69.6)	506 (71.2)	448 (62.3)
Read & write only	83 (3.9)	32 (4.5)	35 (4.9)	16 (2.2)
Primary education	521 (24.3)	165 (23.0)	128 (18.0)	228 (31.7)
Secondary and above	90 (4.1)	21 (2.9)	42 (5.9)	27 (3.8)
Marital status, *n (%)*				
Married	2064 (96.1)	684 (95.4)	687 (96.6)	693 (96.4)
Single	4 (0.2)	0 (0)	0(0)	4 (0.6)
Widowed/divorced	79 (3.7)	33 (4.6)	24 (3.4)	22 (3)

^1^ Values are means ± SDs or n (%). CORN, Community Based Reproductive Nurses;

^2^ USD ~19.89 ETB at the time of data collection

HP, Health Post; HC, Health Center

#### Effect of CORN interventions on SBA utilization

In just nine months of intervention, with the deployment of CORN to intervention arms, the overall SBA utilization rate increased by 81.1% (from 24.61 to 44.59) and 122.9% (from 16.41 to 36.59) in the HP and HC based intervention arms, respectively. Conversely, a slight decline (2%) was observed in the control villages. This yielded almost an equal 21.32 and 20.52 percentage points (PP) increase for the two intervention arms compared to the baseline and control arm (p <0.001). SBA at home by skilled provider has also increased by 1.22 PP in the HP-based intervention arm; while declined by 2.86 PP in the HC based intervention arm (Table 2). On the other hand, compared to the control and baseline, home deliveries decreased by 21.16 PP and 18.5 PP in the HP and HC-based intervention villages, respectively, (p<0.05), Table 2.

**Table 2:** Utilization of SBA services and home delivery at baseline and end line of the study in rural Gedeo, Southern Ethiopia.

Variable	Control # (%)	CORN HP # (%)	DID	p value	CORN HC # (%)	DID PP	p value
Mothers, n (baseline/end line)	717/715^1^	711/722			719/705		
SBA at home							
Baseline	16 (2.23)	21 (2.95)			34 (4.73)		
End line	8 (1.11)	22 (3.05)	1.22	0.035*	6 (0.85)	-2.86	0.018*
SBA at HP							
Baseline	0 (0)	0(0)			0 (0)		
End line	0 (0)	179 (24.79)	24.79	0.001*	55 (7.80)	7.80	0.01*
SBA at HI^†^							
Baseline	193 (26.91)	154 (21.66)			84 (11.68)		
End line	198 (27.69)	121 (16.76)	-5.65	0.065	197 (27.94)	15.48	0.02*
SBA Total							
Baseline	209 (29.15)	175 (24.61)			118 (16.41)		
End line	206 (28.81)	322 (44.59)	20.32	< 0.001*	258 (36.59)	20.52	<0.001
Home delivery^2^							
Baseline	508 (70.85)	536 (75.38)			601(83.58)		
End line	515 (72.03)	400 (55.40)	-21.16	< 0.001*	474 (67.23)	-18.5	0.03*

* Statistically significant, DID, Difference in Difference

^†^ Health institutions (Health center, hospital and clinics)

^1^n is for baseline/endline

^2^Includes home deliveries attended by relatives, traditional birth attendants and non-skilled health workers like health extension workers; but not CORN or other skilled professionals.

#### Effect of the interventions on place of delivery

The HC-based intervention improved health center based deliveries from 8.76 to 25.25 during the nine months intervention period with a net 16.43 PP increase as compared to the baseline and control arm (p<0.001). Similarly, delivery in the health posts increased from 0.14 to 24.79 in the HP-based intervention villages yielding an increase of 21.2 PP compared to the control arm (p<0.001). The overall home deliveries (attended by both skilled and non-skilled attendants), declined by 15.60 and 17.80 percentage points in the HC and HP-based intervention villages, respectively (Table 3).

**Table 3:** Mothers place of delivery, at baseline and end line of the study in rural Gedeo, Southern Ethiopia.

Place of delivery	Control # (%)	CORN HP # (%)	DID	p value	CORN HC n (%)	DID	p value
Mothers, n	717/715^1^	711/722			719/705		
Hospital							
Baseline	39 (5.44)	48 (6.75)			19 (2.64)		
End line	40 (5.59)	33 (4.57)	-2.33	0.72	90 (12.76)	9.97	0.87
Health Center							
Baseline	151 (21.06)	103 (14.48)			63 (8.76)		
End line	151 (21.12)	81 (11.21)	-3.33	0.87	178 (25.25)	16.43	<0.001**
Clinics							
Baseline	3 (0.42)	3 (0.42)			8 (1.11)		
End line	7 (0.98)	7 (0.97)	0.00	0.99	16 (2.27)	-0.3	0.92
Health Post							
Baseline	11 (1.53)	1 (0.14)			12 (1.67)		
End line	36 (5.03)	179 (24.79)	21.16	<0.001	55 (7.80)	2.63	0.05*
Home^2^							
Baseline	513 (71.55)	556 (78.19)			623 (86.65)		
End line	481 (67.27)	422 (58.45)	-15.6	0.01*	453 (64.26)	-17.8	<0.001

* Statistically significant, p< 0.05^,^

** Highly statistically significant, p < 0.001;

DID, Difference in Difference

^1^n is for baseline/endline

^2^Includes all home deliveries attended by skilled and non-skilled professionals including health extension workers

## Discussion

In the present study, we aimed to determine the effect of an innovative community based approach applied to a grass root level in rural Ethiopia on SBA service uptake; i.e. effect of deploying trained community based nurses on the uptake of SBA services. Accordingly, we observed that CORN deployed to rural villages (both stationed at HP or HC) significantly improved SBA uptake by about 20 percentage points. To the best of our knowledge, this is the first study to evaluate the effect of community-based intervention [43] through skilled nurses on the coverage of SBA in low income settings.

Evidence is accumulating on the fact that most maternal deaths and disabilities could be prevented if women have access to good quality health services during pregnancy and childbirth [43–45]. However, many women in low income settings, due to reasons related to geographic, social, cultural, economic, and psychological or other reasons; don’t have access to such services. A more recent estimates revealed that just half of all deliveries in LMICs take place with a skilled attendant, with rates in some countries dropping as low as 8% [46, 47].

In addition to lack of good quality, accessible, and safe motherhood services; a range of factors contribute to women’s poor maternal health. Poverty, unequal access to education, low social status, and lack of income and employment opportunity being the most critical [48]. As such, improving SBA utilization rate is a key intervention leading to a significant reduction in maternal and child deaths [12, 47]. This is because, more than two-third of maternal deaths and neonatal deaths occur in the late stage of pregnancy and within 48 hours of delivery [7,49]. In the same way, more than a third of these maternal deaths can be avoided through the prevention of major complications such as obstructed labor, eclampsia, sepsis and obstetric hemorrhage by SBA [9,50].

On the other hand, evidences from literature involving community-based studies shows that SBA and other community-based intervention could reduce child mortality very significantly [3, 48]. A good example of such evidence is the evidences from countries like Malaysia and Srilanka; whereby governments’ investment to the expansion of community-based midwifery personnel resulted in a rapid shift in the country’s rural health services system [51].

In Ethiopia; the barriers to access quality SBA services, not only overlap with the rest of the world, but also extends far with additional conditions. Socio-economic and gender related factors including distance, low educational status, and lack of women’s autonomy and others are major drivers of the low SBA utilization rate [52–55]. With prevailing barriers and absence of adequate trained human resource for health, Ethiopia may face serious challenge to meet its ambitious target of achieving SBA rate of 90% by 2020 [33].

Today, Ethiopia mainly relies on the HEP to improve maternal and child health at community level. Nevertheless; in spite of HEP’s contribution to improvement of women’s utilization of family planning, antenatal care (ANC) and HIV testing, its contribution to improve health facility delivery and postnatal care has been insignificant [30,56]. Understanding such limitations and skill gaps as well as shortage of facilities for skilled birth at health post level, the federal ministry of health in Ethiopia also recommends institutional (health center or higher) deliveries for SBA.

Conversely, the present study foreshadows a promising strategy to improve SBA and other related maternal health services through the deployment of trained nurses in hard-to-reach communities. Based on our finding, deployment of contextually trained nurses to the rural and hard-to-reach communities and rural kebeles may fill this gap and can have several additional advantages for the HEP. Future implementation of the new approach could also contribute for national policy to effect the Sustainable Development Goals (SDG) that targets reducing maternal mortality rates to 70/100,000 live births by 2030 [2].

The newly proposed health cadres for the health post, CORN, in addition to providing skilled and a better quality maternity services to the unreached rural communities, they could help in coaching, mentoring and supervising the health extension workers. They could also strengthen the overall functionality of the HEP though better managerial skills and lowering tier job burden of HEP. The good news is, both the national and regional level governments has ratified an ambitious plan to expand HEP services as well as the scope. The plan entails availing additional equipment and facilities for skilled delivery and other maternity services as well as basic curative services to rural communities through HEP.

The study had some limitations to consider while interpreting the results. Firstly, even if attempts were made to keep a buffer zone between villages across the study arms during cluster selection and randomization, this might not be ensured during implementation as some families might have shifted during the process. Second, CORN identified pregnant mothers at home during various stages of pregnancy making early identification and enrollment of pregnant women inconsistent, which definitely affect the follow up and the decision to delivery in health facilities. Third, cost effectiveness analysis was not performed in comparison with the HEP. These shortfalls were largely because of financial constraints that limited the duration of the program and the depth as well as scope of the study, which led to investigating the effectiveness than quality and cost-effectiveness. Fourth, even if study arms were randomized and differences in baseline characteristics has limited effect on the result, mothers in the HC-based intervention villages had significantly better educational status compared to HP-based intervention and the control villages.

The Federal ministry of health of Ethiopia is running a level IV HEWs, who would provide skilled maternity care (ANC, DC and PNC) at the health post level. As most of the core competencies that Level IV HEW had are similar to the CORN, evidence generated from this implementation study is expected to support decision making and direction of regional and federal governments with regard to training and deploying CORN in an effort to reduce maternal and neonatal mortality in the country.

### Conclusion

This community-based intervention through trained nurses has improved the coverage of SBA by more than 80% in rural population of resource limited settings. This approach could be scaled in similar settings during the SDG era to improve the health of mothers and newborns. As the intervention could also have a potential long-term effect on strengthening the health system at grass root level, national and regional level governments should consider strengthening the HEP by deploying community based skilled nurses with reproductive health and other related competencies. Additional studies in different communities in agrarian, pastoralist and urban settings are needed to evaluate the cost-effectiveness and long-term effects of deploying trained nurse at the health post level where the foundation of primary health care is lied/underway in the country.

## Supporting information

S1 Data (ZIP)
